# The Role of mHealth Applications in Uro-Oncology: A Systematic Review and Future Directions

**DOI:** 10.3390/cancers17162613

**Published:** 2025-08-09

**Authors:** Miguel Ángel Gómez-Luque, Inés Rivero-Belenchón, Carmen Belén Congregado-Ruiz, German Antonio Escobar-Rodríguez, Francisco Javier Delgado-Granados, Jose Antonio Rivas-González, Rafael Antonio Medina-López

**Affiliations:** 1 Urology and Nephrology Department, Virgen del Rocio University Hospital, 41013 Seville, Spain; ines.rivero.belenchon@gmail.com (I.R.-B.);; 2 Biomedical Institute of Seville (IBiS), Consejo Superior de Investigaciones Científicas (CSIC), University of Seville, 41013 Seville, Spain; 3 Innovation and Technology Group, Biomedical Institute of Seville (IBiS), 41013 Seville, Spain

**Keywords:** eHealth, uro-oncology, prostate cancer, patient empowerment

## Abstract

**Purpose**: This systematic review aims to evaluate the utility of mobile health (mHealth) applications in uro-oncology, hereafter referred to as mHealth apps, specifically examining their potential to improve patient care, symptom management, and communication in genitourinary cancer treatment. **Methods**: Adhering to the Preferred Reporting Items for Systematic Reviews and Meta-Analyses (PRISMA) guidelines, a comprehensive systematic review was conducted focusing on mHealth applications for patients with genitourinary cancers. **Results**: The review analyzed 29 studies, which revealed that mHealth apps demonstrated potential in uro-oncology patient care. Key findings included effective symptom monitoring, enhanced decision support, and improved patient education. The applications were found to be feasible and well-accepted by patients. However, implementation challenges were identified, including technical barriers, variations in app quality, and unequal access to digital healthcare technologies. This review systematically categorized mHealth interventions into three functional domains—symptom management, decision support, and personalized care—and identified critical implementation barriers including digital inequity, high risk of bias, and app quality variability. **Conclusions**: Mobile health applications demonstrate promise in revolutionizing uro-oncology care. Future research should prioritize developing comprehensive applications that address a broader range of urological cancers, enhance patient–clinician communication, and undergo rigorous evaluation. Collaborative efforts among researchers, clinicians, and app developers are crucial to overcome existing limitations and maximize the potential of these innovative healthcare tools.

## Simple Summary

This systematic review explores how mobile health applications can improve care for individuals with cancers affecting the urinary system, including prostate, bladder, and kidney cancers. We aimed to understand whether these applications effectively assist in managing symptoms, educating patients, and enhancing communication with healthcare professionals. Our analysis of 29 studies revealed that these applications show promise in monitoring patient symptoms, aiding in decision-making, and providing personalized care. Patients generally found these tools acceptable and easy to use. However, challenges such as unequal access to technology, difficulties in app utilization, and variable application quality were identified. We conclude that mobile health applications could significantly advance cancer care, but future development must prioritize widespread accessibility, consistent quality, and broader coverage for various cancer types to truly improve patient outcomes and experiences.

## 1. Introduction

Information and communication technologies (ICT) represent a new opportunity to improve healthcare, as they offer patients and practitioners new methods of improving overall health, such as novel ways of monitoring chronic diseases and access to healthcare. The World Health Organization (WHO) defines eHealth as “the use of ICT for health” [1]. The breadth of this definition has led to the term eHealth involving many different uses, from the infrastructure to access imaging tests to teleconsulting implementation or augmented reality [2]. However, with the increasing number of apps (included in the umbrella term “mHealth”), several concerns arise, such as their actual impact on monitoring symptoms [3], the possible lack of privacy and personal data security, or the inequalities in access to technology between different groups of people [4].

As any other medical specialty, urology may benefit from the advances in this field. Genitourinary cancer represents a fundamental challenge for healthcare systems globally, accounting for 25% of all cancer diagnoses [5]. Together with the advances in diagnosis and treatment that have improved overall survival, patients with urologic cancer face various treatment side effects that require careful management. Outpatient care has become the norm, increasing the need for tools that facilitate communication between patients and healthcare professionals, symptom self-management, patient decision-making, and support for care [6,7]. In this context, mobile health (mHealth) applications have emerged as a promising tool for improving urologic cancer care [8]. The growing popularity of smartphones, even among older adults [9], has driven the development of apps targeted at cancer patients, including those focusing on genitourinary tumors [10].

Recent reviews have examined digital health tools and mHealth interventions in oncology, especially within the scope of prostate cancer or broader cancer care [10,11,12]. However, these publications tend to focus on app evaluation metrics, detection or screening strategies, or cancer types in isolation. To our knowledge, this is the first systematic review that categorizes mHealth interventions in uro-oncology into distinct functional domains—symptom management, decision support, and personalized care—while also providing an implementation-oriented synthesis across multiple genitourinary malignancies.

This systematic review will focus on evaluating the existing evidence on the use of mHealth apps in the uro-oncology setting. It will analyze the types of available apps, their features, the quality of the evidence supporting their effectiveness, and the challenges faced in their implementation and use. The aim is to address three key questions: (1) What is the role of mHealth applications in uro-oncology care? (2) What is the effectiveness of mHealth applications in improving patient outcomes, symptom management, and quality of life? (3) What are the main challenges and barriers to the implementation of these tools in clinical practice?

## 2. Material and Methods

### 2.1. Study Design

This systematic review was designed to assess (1) the role, (2) the effectiveness, and (3) the challenges of mobile health (mHealth) applications in uro-oncology. We adhered to the Preferred Reporting Items for Systematic Reviews and Meta-Analyses (PRISMA) [13] to ensure a transparent and reproductible evaluation of the evidence. The primary objective is to examine the utility of mHealth apps in monitoring, managing symptoms, improving communication, and enhancing care in patients with urologic cancers, including prostate, bladder, and kidney cancers. This systematic review was prospectively registered in PROSPERO (ID: 1120942) to ensure methodological transparency and to minimize potential reporting bias. The protocol is available on the PROSPERO database.

### 2.2. Eligibility Criteria

We included peer-reviewed studies of adults with prostate, bladder, or kidney cancer that evaluated smartphone-based interventions. Comparators were not required. Outcomes of interest comprised treatment efficacy, symptom management, patient adherence, quality of life, and satisfaction. We excluded grey literature, non-English publications, case reports, editorials, and conference abstracts.

### 2.3. Search Strategy

This review prioritized high-quality evidence by focusing on peer-reviewed articles, clinical trials, cohort studies, case–control studies, and qualitative and observational studies. Searches were conducted from 14 March 2025 to 31 March 2025.

A systematic search was conducted including studies published up to March 2025 using two databases: PubMed and Web of Science (WoS). The search was designed to capture all relevant studies on mHealth applications in uro-oncology. We selected PubMed and Web of Science due to their extensive coverage of biomedical and clinical research, which was appropriate for the focus on uro-oncological interventions. While others may include valuable technological literature, we prioritized databases that emphasize clinical validation, patient outcomes, and peer-reviewed medical studies, consistent with PRISMA guidelines. The search terms used in both databases were as follows:

(“Prostatic Neoplasms” [MeSH Terms] OR “Urinary Bladder Neoplasms” [MeSH Terms] OR “Kidney Neoplasms” [MeSH Terms] OR “uro-oncology” OR “urological cancers” OR “prostate cancer” OR “bladder cancer” OR “kidney cancer”)

AND

(“Mobile Applications” [MeSH Terms] OR “mHealth” OR “mobile health apps” OR “mobile apps” OR “smartphone applications”)

AND

(“Treatment Outcome” [MeSH Terms] OR “Patient Compliance” [MeSH Terms] OR “outcome” OR “impact” OR “adherence” OR “symptom management”)

### 2.4. Selection Process

Two independent reviewers (MAGL, IRB) screened the titles and abstracts of all the identified studies to determine eligibility. Any disagreements were resolved through discussion, and if consensus could not be reached, a third reviewer (CBCR) was consulted. Full texts of the selected studies were retrieved, and eligibility was confirmed by applying the inclusion and exclusion criteria.

In cases where studies presented conflicting results or overlapping populations, inclusion was based on the methodological quality, recency, and relevance to the predefined outcomes.

### 2.5. Data Extraction

Data were extracted using a predefined template, which included the following:

- Study characteristics: year of publication, study design, and sample size.
- Participant characteristics: type of cancer and stage (localized, locally advanced, metastatic) and age range.
- Intervention details: type of mHealth application, features, and functionality.
- Comparators: description of control or comparison interventions (if applicable).
- Key findings related to treatment outcomes, symptom management, adherence, quality of life, and patient satisfaction.

### 2.6. Data Synthesis

Due to the anticipated heterogeneity of the interventions and outcomes, a narrative synthesis will be used to summarize the findings. Although the dataset included some randomized controlled trials, the considerable heterogeneity in populations, interventions, and outcome measures precluded a meaningful quantitative synthesis. Heterogeneity was addressed descriptively by classifying studies into thematic domains based on intervention purpose and clinical focus, allowing for structured synthesis despite methodological differences. Therefore, a narrative synthesis approach was applied. Key themes to be identified included the following:

- Types of mHealth applications used in uro-oncology.
- Reported benefits in terms of patient outcomes and care quality.
- Barriers to the effective implementation of mHealth technologies.

### 2.7. Risk of Bias Assessment

The internal validity and risk of bias for each eligible study were formally assessed by two independent reviewers (MAGL, IRB) using standardized Cochrane tools. Any disagreements were resolved through discussion to reach a consensus, with a third reviewer (CBCR) available for arbitration if needed.

For randomized controlled trials (RCTs), the revised Cochrane Risk-of-Bias tool 2 (RoB 2) was applied [14]. This tool evaluates bias across five domains: (1) bias arising from the randomization process, (2) bias due to deviations from intended interventions, (3) bias due to missing outcome data, (4) bias in measurement of the outcome, and (5) bias in selection of the reported result.

For non-randomized studies of interventions (NRSIs), the Risk of Bias in Non-randomized Studies of Interventions (ROBINS-I) tool was used [15]. It assesses bias across seven domains: (1) bias due to confounding, (2) bias in selection of participants into the study, (3) bias in classification of interventions, (4) bias due to deviations from intended interventions, (5) bias due to missing data, (6) bias in measurement of outcomes, and (7) bias in selection of the reported result.

The overall risk of bias for each study was judged as ‘Low risk’, ‘Some concerns’, or ‘High risk’ for RCTs, and ‘Low’, ‘Moderate’, ‘Serious’, or ‘Critical’ risk for NRSIs.

## 3. Results

**Figure 1:**
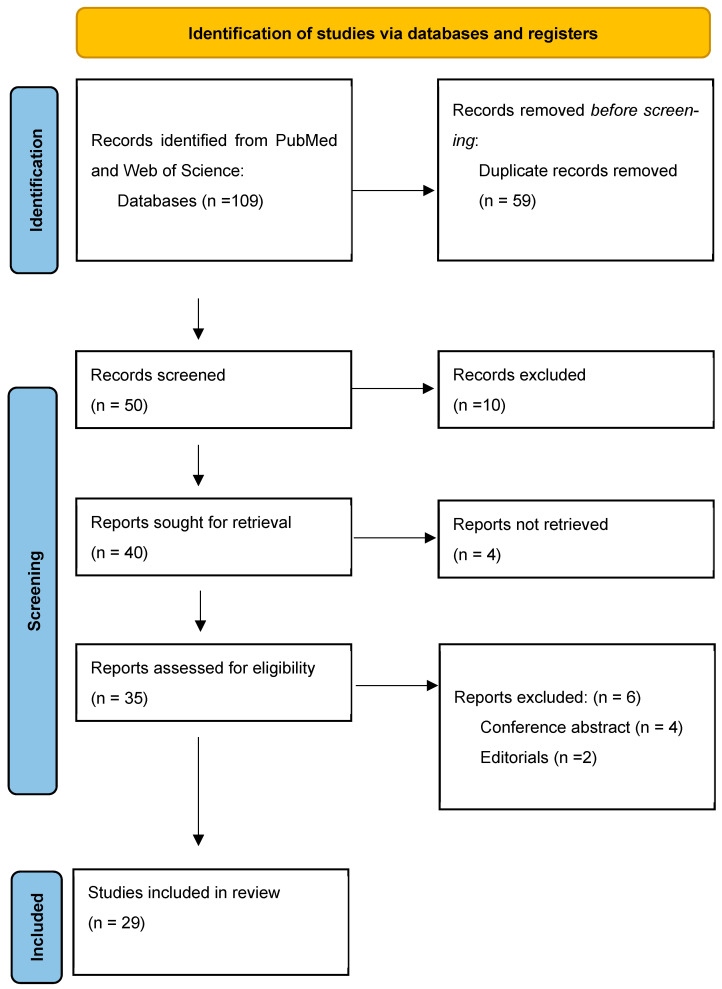
Selection process.

In March 2025, 109 articles were identified: two systematic reviews and meta-analysis, 18 clinical trials, and 89 original articles. After duplicates were removed, 50 articles were screened by title and abstract. Then, 35 records were assessed via screening of the full text. The inclusion criteria were described above. Finally, 29 studies were selected for eligibility for the study analysis, each assessing the application of mHealth technologies in the field of uro-oncology. A structured overview of the review process and article selection pathway is provided in Figure 1, following the recommended flowchart of the PRISMA guidelines.

This systematic review included studies evaluating mobile applications for symptom monitoring and management in patients with urological cancer. The majority of studies focused on prostate cancer, with a limited number of studies including patients with other types of urological cancer, such as bladder cancer and renal cancer. The studies were conducted across various settings, including university hospitals, oncology centers, and outpatient clinics. A detailed overview of the study characteristics, including the sample size, type of cancer, interventions, comparators, and key outcomes, is provided in Table 1. Most studies employed observational or pilot designs, with a predominant focus on prostate cancer and symptom monitoring functionalities. While individual study results are described in detail, the following synthesis emphasizes cross-cutting themes, patterns, and divergences across studies, particularly concerning the clinical impact of mHealth applications in uro-oncology.

The study designs varied, comprising feasibility studies, randomized controlled trials, qualitative studies, and observational studies. Sample sizes showed considerable variation, ranging from small-scale feasibility studies with fewer than 10 participants to randomized controlled trials involving more than 100 participants.

The evaluated interventions encompassed various mobile applications with distinct features, including symptom logging, access to evidence-based self-care advice, alert systems to notify healthcare professionals about severe symptoms, and tools for data tracking and analysis.

The outcome measures assessed in the studies included symptom burden, quality of life, patient–healthcare provider communication, patient satisfaction with the application, and healthcare service utilization.

### 3.1. Role of mHealth Interventions

The mHealth interventions examined in this review can be broadly categorized into three main areas: (1) symptom monitoring, (2) decision-support and educational tools, and (3) personalized care approaches.

Based on the review of the literature and the most common objectives addressed in the included studies, we identified these three primary areas as the ones in which mHealth interventions have shown promising potential to impact uro-oncological patient care. These domains were chosen because they directly align with critical components of cancer management, aiming to enhance clinical outcomes, patient empowerment, and overall quality of care.

#### 3.1.1. Symptom Monitoring and Management

The implementation of mobile health (mHealth) applications has proven to be a promising tool for symptom monitoring and management in patients with genitourinary cancer. The reviewed studies indicate that these interventions are feasible and highly accepted by patients.

Regarding adherence, daily symptom reporting through interactive applications, such as Interaktor, showed high compliance rates [16]. In prostate cancer patients undergoing radiotherapy, the average adherence was 87% (median 92%), and it was 83% in breast and prostate cancer patients. High usability and a satisfaction rate exceeding 80% were observed with personalized applications for preparation and recovery after radical prostatectomy [17].

Concerning clinical impact, app-based interventions demonstrated noteworthy improvements in symptom burden and quality of life. Specifically, in prostate cancer patients undergoing radiotherapy, lower levels of fatigue and nausea were reported at the end of treatment, as well as a reduced burden of emotional functioning, insomnia, and urinary symptoms both at treatment completion and three months post-treatment [18]. The Interaktor application, in particular, was associated with a reduction in symptom burden, especially urinary symptoms and those related to emotional functioning. Improvements in the irritative and obstructive urinary domains of quality of life were also reported in prostate cancer patients receiving androgen deprivation therapy [19]. Furthermore, some applications improved psychological well-being and self-care abilities in post-radical prostatectomy patients, contributing to an overall better quality of life [20].

Key functionalities of these applications typically include symptom assessment (occurrence, frequency, discomfort level) [21], an alert system that notifies healthcare professionals about severe symptoms, continuous access to evidence-based self-care advice, visualization of symptom history through graphs, and a free-text function for additional comments.

**Table 1:** Overview of included studies evaluating mHealth applications in uro-oncology.

Study and Year	Study Design	Sample Size	Age Range	Urological Malignancy	Study Intervention	Comparator	Key Findings
Amor-García et al., 2020 [10]	Cross-sectional descriptive observational study	46 smartphone applications	N/A (mHealth app study)	Genitourinary malignancies (prostate, testicular, bladder, kidney, cervical, ovarian, uterine, endometrial, vulva)	Evaluation of mobile health (mHealth) application quality for patients	N/A (descriptive study)	Avg. MARS score 2.98; most apps informative; “Engagement” domain scored lowest.
Belarmino et al., 2018 [22]	Qualitative usability study	Not specified	N/S	Prostate cancer (post-robot-assisted radical prostatectomy)	Mobile health application to monitor recovery and patient-reported outcomes	N/A (usability study)	App was feasible/usable; high questionnaire response/compliance rates for post-op activities.
Bergerot et al., 2025 [23]	Longitudinal pilot clinical trial	50 patients	Median 59 years (range: 32–88)	Metastatic renal cell carcinoma (mRCC)	Mindfulness application (CARINAE)	No control group specified for causality	App feasible/acceptable; improvements in emotional symptoms, fatigue, mindfulness, and HRQoL.
Blair et al., 2021 [24]	Pilot randomized controlled trial (RCT), mixed-methods	44 participants in intervention arm	Mean 63 years (SD 11; range: 40–85)	Prostate cancer (41%), also breast and colorectal cancer	App-based brisk walking intervention	Control group with usual care	Intervention feasible/acceptable; health coaching group showed increase in daily steps/moderate-intensity physical activity.
Camacho-Rivera et al., 2019 [25]	Cross-sectional observational study	473 residents	Over 18 years old	Prostate and colorectal cancer screening	Evaluation of smartphone and health app use	N/A (descriptive study)	No noteworthy differences in smartphone/health app access across age groups; education predicted health app access.
Carhuapoma et al., 2021 [26]	Pre-test/post-test design, with qualitative and quantitative methods	Target of 158 patient-decision partner dyads	N/S	Prostate cancer	Multicomponent mHealth decision aid intervention to facilitate partner involvement	Enhanced usual care (EUC) group	Protocol for RCT evaluating mHealth decision aid on HRQL-PSY, decision conflict, regret.
Crafoord et al., 2020 [21]	Mixed-methods study	75 prostate cancer patients, 74 breast cancer patients	Prostate cancer: median 72 years (range 44–81)	Prostate cancer (also breast cancer)	Interaktor interactive app for symptom self-management	N/A (descriptive study of use and perception)	High adherence to daily symptom reporting; app perceived as easy to use and supportive for self-care.
Crafoord et al., 2025 [27]	Two parallel, open-label randomized controlled trials (RCTs)	75 intervention group and 75 control group for prostate cancer (P-RCT)	N/S	Prostate cancer (P-RCT), also breast cancer (B-RCT)	Interaktor interactive app for patient-reported outcomes (ePRO) and interactive support	Control group with standard care	Interaktor reduced QALYs loss at low cost; ePROs associated with lower symptom burden; nurses reported no increased workload.
Hälleberg Nyman et al., 2017 [28]	Qualitative descriptive study, part of an experimental study	28 patients (17 used the app)	57 to 77 years	Prostate cancer	Interaktor app for daily symptom reporting and self-care	Historical control group with standard care	App users experienced more mutual care participation, felt more active, and had continuous health service contact.
Jin et al., 2024 [29]	Non-randomized pilot feasibility and acceptability study	18 patients	N/S	Prostate cancer (undergoing radiotherapy)	Smart water bottle and app (HidrateSpark 3) to improve bladder filling adherence	Retrospectively matched controls (compliance data in intervention arm only)	Bladder filling compliance met; high patient engagement (83% used >50% treatments).
Kelmendi et al., 2024 [30]	Single-arm, descriptive feasibility study (qualitative and quantitative)	11 patients	Range 57 to 75 years (mean 66, median 68)	Prostate cancer	Complex intervention with ePROs, self-management advice in an app, and nurse support in primary care	N/A (single-arm study)	Nurse support + app intervention feasible in prostate cancer patients; valued personalized support; high app symptom reporting adherence.
Kennedy et al., 2025 [31]	Mixed-methods study with embedded design, part of a pilot RCT	44 participants in intervention arm	Mean 63 years (SD 11; range 40–85)	Prostate cancer (41%), also breast and colorectal cancer	App-based brisk walking intervention (APROACH)	Control arm with usual care	Behavioral support intervention showed high fidelity in BCT delivery; app useful for habit formation, but use decreased over time.
Kondylakis et al., 2025 [32]	Feasibility randomized controlled trial (RCT)	50 patients (23 intervention, 27 control), 39 completed trial	Intervention group: mean 45.38 (SD 26.2, range 12–91 years); control group: mean 56.66 (SD 27, range 12–91 years)	Prostate cancer (three in intervention, two in control), bladder cancer (two in intervention, four in control); also includes cardiac and orthopedic surgeries	CARINAE digital solution for perioperative stress and anxiety reduction	Control group with standard care	CARINAE feasible for stress/anxiety management; trend for lower stress; difference in HADS depression in one hospital; provider involvement crucial.
La Rocca et al., 2025 [33]	Cross-sectional descriptive observational study	10 mobile health applications (MHAs)	N/A (app study)	Bladder cancer	Review and evaluation of MHAs for bladder cancer	N/A (descriptive study)	MHAs for BCa showed suboptimal quality (low MARS scores); less than one-third adhered to EAU guidelines; 100% covered BCa definition/treatment.
Lai et al., 2024 [12]	Retrospective genetic analysis of CAFs-RGs and predictive nomogram construction	554 samples (386 PCa, 52 normal adjacent) plus data from 199 and 248 PCa patients from public databases	N/S	Prostate cancer	Development of a nomogram to predict clinical outcome and radiotherapy prognosis	N/A (predictive model development study)	Identified CAFsRGs predicting PCa prognosis/radiotherapy response; developed high-accuracy nomogram/online app for BRFS.
Langius-Eklöf et al., 2017 [16]	Prospective, randomized, controlled trial	For prostate cancer, related study had 66 intervention and 64 control	Over 18 years old	Prostate cancer	Interaktor interactive application for daily symptom reporting and self-care	Control group with standard care	Protocol for RCT evaluating Interaktor’s effect on symptom burden, QoL, health literacy, disease progression, costs.
Langius-Eklöf et al., 2017 [34]	Description of logged data and interviews, compared with a historical control group	66 patients; 53 interviewed	Mean age 69 years	Prostate cancer	Interaktor interactive application for symptom management during radiotherapy	Historical control group	High adherence (87%); app easy to use, provided security; facilitated self-management/person-centered care.
Lee et al., 2024 [19]	Randomized, single-blind, waiting-list controlled trial	48 patients (24 experimental, 24 control); 46 included in final analysis	Mean age 68.83 (SD 7.09) years	Prostate cancer	4-week nurse-led mobile program on exercise and diet	Waiting-list control group with usual care	Program improved MetS components (glucose, abdominal circumference), body composition; significant effect on irritative/obstructive urinary HRQoL.
Martini et al., 2024 [17]	Prospective non-randomized study	122 patients (62 in optimized pathway, 60 in standard care)	64–65 years	Prostate cancer (post-radical prostatectomy)	Optimized perioperative program with a personalized mobile application for preparation and recovery	Standard of care (SOC) group	App-based program improved 6-week continence rate (92% vs. 75%, *p* = 0.01); fewer grade ≥ 2 complications; increased same-day discharge; high usability/satisfaction.
Mohseni et al., 2023 [8]	Two-phase app development and usability evaluation	Phase 1: 15 specialists; Phase 2: 21 patients, 10 specialists	Specialists: mean 44.90 ± 3.51 years; patients: not specified.	Prostate cancer	Development of a mobile application for electronic patient-reported outcomes	N/A (development and usability evaluation study)	App for ePROs/side effect reporting developed; high satisfaction among patients/specialists; app functions deemed necessary.
Nabi et al., 2020 [35]	Qualitative usability study with focus groups and in-depth interviews	Five patients, five physicians	Patients: mean 62 years (range 45–75)	Prostate cancer	Evaluation of an mHealth mobile application (name not specified)	N/A (usability study)	Patients appreciated holistic care; registration difficulties (60%); physicians underestimated patient tech ability; patients comfortable documenting exercise/diet.
Obro et al., 2022 [36]	Qualitative usability study, with individual and group interviews	Four urological nurses and one physician; patient number not specified	Nurses: between 30 and 52 years; physician: not specified	Low risk localized prostate cancer	19-week mHealth coaching program	N/A (qualitative usability study)	Nurses found coaching increased autonomy/attentiveness; lack of mHealth competencies reduced motivation.
Peng et al., 2024 [20]	Retrospective study, with assignment by clinical eligibility	112 patients (56 per group)	Older men, exact range not specified	Prostate cancer (post-radical prostatectomy)	Mobile internet management for continuous care	Control group with standard care	Mobile internet management improved patient knowledge, emotional well-being, and self-care abilities.
Pereira-Azevedo et al., 2018 [11]	Review article	N/A (review)	N/A (review)	Prostate cancer	Discussion on eHealth and mHealth in detection and active surveillance (e.g., risk calculators, monitoring apps)	N/A (review)	eHealth market growing but underutilized; RPCRC, PRIAS, Follow MyPSA are value-added tools.
Roman Souza et al., 2024 [37]	Pilot clinical trial	20 patients	Range 49 to 82 years, mean 66 years (SD 11)	Stage IV renal cell carcinoma (RCC)	Mobile health (mHealth) application for education and symptom management (educational modules and algorithm)	N/A (feasibility and acceptability study)	App met acceptability/feasibility criteria; knowledge test score changes after educational modules.
Sundberg et al., 2015 [38]	Feasibility study	Nine patients	Mean age 69 years	Prostate cancer	Interactive ICT platform (mobile application) for symptom assessment and management	N/A (feasibility study)	App feasible/acceptable; relevant questionnaire/self-care advice; alerts led to nurse contact/support; facilitated patient participation/communication.
Sundberg et al., 2017 [18]	Non-randomized controlled study (historically controlled)	130 patients (66 intervention, 64 control)	Mean 69 years (range 52–82)	Prostate cancer	Interaktor interactive application for early detection, reporting, and symptom management	Control group with standard care	Intervention group had lower fatigue/nausea; reduced emotional functioning, insomnia, urinary symptoms; app facilitated real-time communication.
Sundberg et al., 2021 [39]	Quasi-experimental design, with historical control group	130 patients (66 intervention group, 64 control group)	Targeted population “middle-aged or older men”	Prostate cancer	Interaktor application for symptom reporting and self-care support	Control group with standard care	Intervention group showed improvements in advanced health literacy skills.
Tran et al., 2020 [40]	Single-arm pilot feasibility trial	29 patients analyzed (out of 30 consented)	Median 55 years	Prostate cancer	Digital health application (Strength Through Insight) for collecting ePROs	N/A (single-arm study)	App feasible (86% satisfactory completion); patients reported ease of use, preference for text messages, increased symptom awareness.

Abbreviations (as they appear in Table 1): N/A (not applicable); MARS (Mobile Application Rating Scale); N/S (not specified); mRCC (metastatic renal cell carcinoma); HRQoL (health-related quality of life); EUC (enhanced usual care); HRQL-PSY (psychosocial health-related quality of life); ePRO (electronic patient reported outcomes); QALYs (quality-adjusted life years); SD (standard deviation); BCT (behavior change technique); HADS (Hospital Anxiety and Depression Scale); MHA (mobile health applications); BCa (bladder cancer); EAU (European Association of Urology); CAFs-RGs (cancer-associated fibroblast-related genes); PCa (prostate cancer); BRFS (biochemical recurrence-free survival); MetS (metabolic syndrome); SOC (standard of care); RPCRC (Rotterdam Prostate Cancer Risk Calculator); PRIAS (Prostate Cancer Research International Active Surveillance); RCC (renal cell carcinoma).

#### 3.1.2. Decision-Support and Educational Tools

Mobile health (mHealth) tools and electronic patient-reported outcomes (ePROs) applications have demonstrated their utility in uro-oncology for decision support and patient education.

In the realm of decision support, the CHAMPION intervention, an mHealth-based application for patients with advanced prostate cancer and their decision partners, showed potential for reducing decisional conflict and improving psychosocial health-related quality of life (HRQoL-PSY). This platform incorporated tutorials on the decision-making process, graphical summaries of HRQoL, and tools for values clarification [26]. Similarly, applications like the Rotterdam Prostate Cancer Risk Calculator (RPCRC) were identified as tools informing decision-making in the reduction in the overdiagnosis and overtreatment in urology [25].

Regarding educational and self-management tools, the Interaktor application proved effective in symptom management for cancer patients, offering self-assessments, an alert system for healthcare professionals, access to evidence-based self-care advice, and symptom history visualization [21]. Adherence to daily symptom reporting with Interaktor was notably high (83% in studies including breast and prostate cancer patients). Patients considered the self-care advice valuable and applicable. Interaktor use also improved advanced health literacy, including the ability to select and evaluate health information. A nurse-led mobile program for prostate cancer patients receiving androgen deprivation therapy (ADT) improved metabolic syndrome components, body composition, and reduced ADT side effects, particularly irritative and obstructive urinary symptoms [19]. Additionally, internet-based mobile management for post-radical prostatectomy patients improved disease knowledge, psychological well-being, and self-care abilities [20]. The integration of an application with a smart water bottle in prostate cancer patients during radiotherapy optimized time in the clinic and linear accelerator [29].

The acceptability of mHealth applications was consistently high, with satisfactory questionnaire completion rates (e.g., 86% in an ePRO study for prostate cancer patients) [40]. Patients often preferred symptom reporting via text messages and found the applications easy to use. The mean Mobile Application Rating Scale (MARS) score for the overall quality of 46 applications was 2.98 (SD 0.77), indicating generally acceptable functionality [10].

#### 3.1.3. Personalized and Targeted Care

Mobile health (mHealth) interventions demonstrate considerable potential for the implementation of personalized and targeted care in oncology. Key personalization mechanisms observed in the literature include adapting the content specifically to individual symptoms, treatment side effects, and lifestyle patterns [19]. The ability to dynamically adjust app content based on patient progression or reported data (e.g., symptoms, activity levels) also contributes to personal relevance and engagement [20], often through predictive analytics and artificial intelligence techniques [32].

These interventions enhance patient empowerment and engagement by promoting self-management, self-monitoring, and self-care activities, increasing awareness of how daily decisions impact health and well-being [16]. Applications provide access to relevant and timely information, improving disease understanding and health literacy [39]. Furthermore, some interventions successfully improved emotional symptoms, fatigue, and quality of life by addressing psychological distress and providing support [20]. The flexibility in patients’ use of app components, according to their individual needs and preferences, was indicative of the tool’s capacity to offer individualized care [28].

### 3.2. Effectiveness of mHealth Interventions

**Figure 2:**
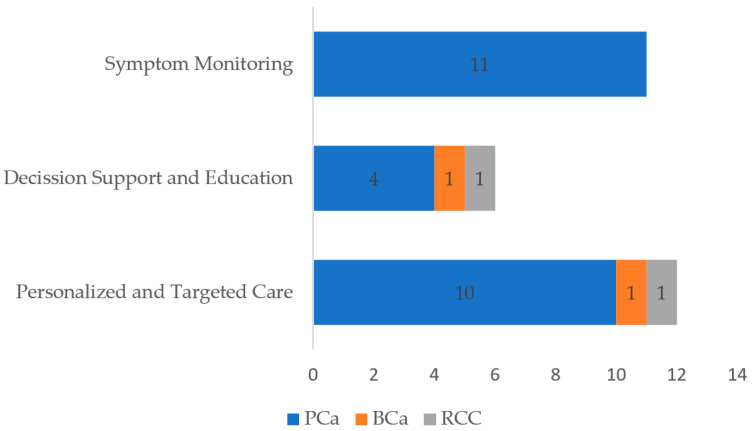
Distribution of studies based on the type of mHealth application. Abbreviations (as they appear in the table): PCa (prostate cancer); BCa (bladder cancer); RCC (renal cell carcinoma).

In order to enhance the clarity and accessibility of the findings, we synthesized the distribution of studies according to the type of mHealth intervention evaluated. As shown in Figure 2, most studies focused on symptom monitoring and personalized and targeted care, followed by decision-support applications. It is important to note that most studies included in this review were conducted in prostate cancer populations, with relatively few addressing bladder or kidney cancer, which limits the generalizability of findings across all uro-oncological contexts.

#### 3.2.1. Symptom Control and Management

Mobile health (mHealth) interventions have proven to be valuable tools for symptom control and management in oncology patients, particularly those receiving outpatient treatment [21].

The observed benefits in symptom control and management are notable. A decrease in levels of fatigue, nausea, emotional distress, insomnia, and urinary symptoms has been demonstrated in patients using these applications [23]. These interventions also improved the health-related quality of life (HRQoL). The real-time monitoring offered by these applications enables early problem detection and timely intervention by healthcare professionals, which can reduce emergency department visits and hospitalizations [34]. Furthermore, these tools foster self-monitoring, active patient participation in their care, and self-care activities. The acceptability and usability of the applications are high, with patients reporting high satisfaction and perceiving them as easy to use and intuitive. Adherence to daily symptom reporting can be high, exceeding 80% in some studies [16].

#### 3.2.2. Patient Engagement and Adherence to Treatment

Mobile health (mHealth) interventions demonstrate a noteworthy impact on fostering patient engagement and adherence to their oncological treatment [10]. High rates of app notification and use were observed; adherence to daily symptom reporting exceeded 80% in studies on prostate and breast cancer patients [21]. In a multinational study with metastatic renal cell carcinoma patients, 75% reported consistent engagement with the application [23]. Patients expressed high satisfaction and perceived the applications as easy-to-use, intuitive, and valuable tools for comprehensive care [35].

App usability and intuitive design were determining factors for adoption and engagement [10]. Applications capable of personalizing content and functions according to individual needs, along with interactive features such as bidirectional communication and data visualization, also enhanced personal relevance and participation. mHealth tools promoted self-monitoring and self-management, increasing awareness of how daily decisions impact health. Furthermore, quality and relevant information, alongside specific functions like daily reminders and alert systems, contributed to engagement [21].

Regarding the impact on treatment adherence and behavior management, mHealth applications improved adherence to diet and exercise recommendations [35]. In the context of radiotherapy for prostate cancer, one application enhanced compliance with bladder filling before treatment [29].

#### 3.2.3. Impact on Quality of Life

Mobile health (mHealth) interventions have demonstrated a notable impact on improving the quality of life (QoL) of oncology patients, addressing various dimensions of physical, emotional, and social well-being [23]. A noteworthy reduction in levels of fatigue and nausea was observed at the end of radiotherapy, as well as a reduced burden on emotional functioning, insomnia, and urinary symptoms both at treatment completion and three months post-treatment [18]. In patients with metastatic renal cell carcinoma (mRCC), significant improvements in emotional symptoms and fatigue were reported [23].

mHealth interventions had a positive impact on patients’ health-related quality of life (HRQoL), including those with prostate cancer [20]. A multinational study with mRCC patients on immunotherapy demonstrated improvements in overall quality of life from baseline to post-intervention (*p* = 0.001 for each outcome) [23]. For prostate cancer patients, although no statistically significant difference in the mean EQ-5DP change between groups was found, the use of the Interaktor application was associated with a lower symptom burden and improved quality of life [27].

In specific QoL dimensions, applications contributed to reducing emotional distress and improving psychological well-being, decreasing anxiety and depression scores. A nurse-led mHealth-based program in prostate cancer patients on androgen deprivation therapy (ADT) significantly improved lifestyle scores (*p* ≤ 0.001), fasting blood glucose (FBS) levels (*p* = 0.05), and abdominal circumference (AC) (*p* = 0.049) [19]. Mobile management also improved patients’ self-care abilities, social function, and mental health [20].

### 3.3. Feasibility and Challenges of mHealth Tools

Mobile health (mHealth) interventions demonstrate notable feasibility in their implementation, although they also face persistent challenges.

Regarding feasibility, mHealth applications have shown high patient acceptance and adherence. Adherence to daily symptom reporting, for instance with Interaktor, has been high, with medians of 83% for daily symptom reporting and 87% in prostate cancer patients during radiotherapy [27]. Studies reported that 96% of patients completed assessments in a mindfulness app-based intervention for metastatic renal cell carcinoma (mRCC) patients, with high adherence rates reported by 75% of patients [23]. A personalized mobile application for preparation and recovery post-radical prostatectomy (RP) showed high usability and satisfaction (>80%) among users [17]. Engagement in using the Interaktor app during radiotherapy for prostate cancer was consistently high, and the use of the smart water bottle app and its companion was feasible with high engagement and acceptance [29]. Mobile technology is widely used and accepted across generations, including older adults. Applications allowing real-time symptom reporting facilitated continuous communication and monitoring by healthcare professionals, generating a sense of security. mHealth also promoted self-management and active patient participation. mHealth interventions have been shown to improve self-care abilities and empower oncology patients [38]. Concerning efficiency, mHealth interventions reduced the time patients spent in the clinic and linear accelerator for treatments, and telemedicine and mHealth were identified as cost-effective alternatives to traditional in-person care [23,29].

However, challenges and limitations persist. Some patients experienced initial difficulties with registration, app download, or internet connection [35]. Device-related issues, such as activity tracker failures or manufacturers discontinuing production, were also identified [24]. Maintaining sustained long-term patient engagement remains a challenge, influenced by patient functional status and app task burden [32]. A crucial concern is the lack of scientific rigor and limited involvement of healthcare professionals in the development of many applications [10,11]. Many applications were merely informative, lacking interactivity, personalized, or evidence-based content. Adherence to clinical guidelines was often low [33]. Skepticism among physicians regarding patients’ ability to use mHealth technology was noted [35]. The privacy and security of sensitive data exchanged over wireless networks raised concerns [11]. Finally, many studies had small sample sizes, focused on specific or highly educated patient populations, and relied on self-reported data, limiting generalizability [16,23,24,25,35].

### 3.4. Risk of Bias of Included Studies

Out of the 29 studies included in the systematic review, 10 were eligible for formal risk of bias assessment based on their interventional design. The remaining 19 studies were not assessed, as their methodologies (e.g., qualitative usability studies, descriptive cross-sectional analyses of application features, or review articles) did not align with the intervention-focused domains of the RoB 2 or ROBINS-I tools. The overall quality of the evidence was variable, with a notable proportion of studies exhibiting methodological concerns.

**Figure 3:**
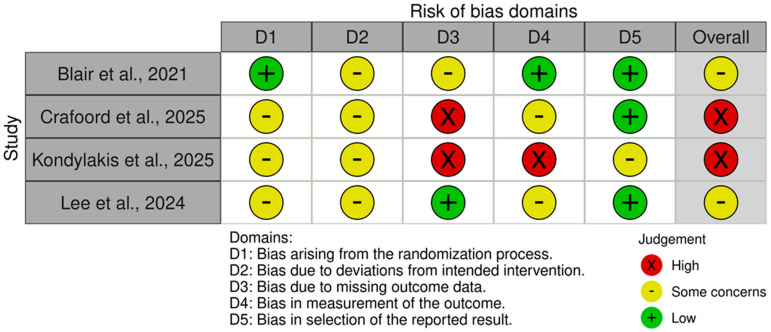
“Traffic light” plots of the domain-level judgement for each individual study evaluated with RoB-2 [19,24,27,32].

**Figure 4:**
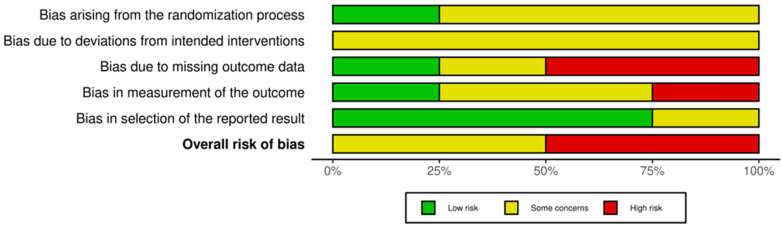
Weighted bar plots of the distribution of risk-of-bias judgements within each bias domain using RoB-2.

The assessment of the four RCTs using the RoB 2 tool is summarized in Figure 3 and Figure 4. Half of the trials (n = 2) were rated as having ‘Some concerns’, the remaining half were deemed to be at ‘High risk’ of bias. Common issues leading to a rating of ‘Some concerns’ or ‘High risk’ were identified in Domain 3 (bias due to missing outcome data) and Domain 4 (bias in measurement of the outcome).

**Figure 5:**
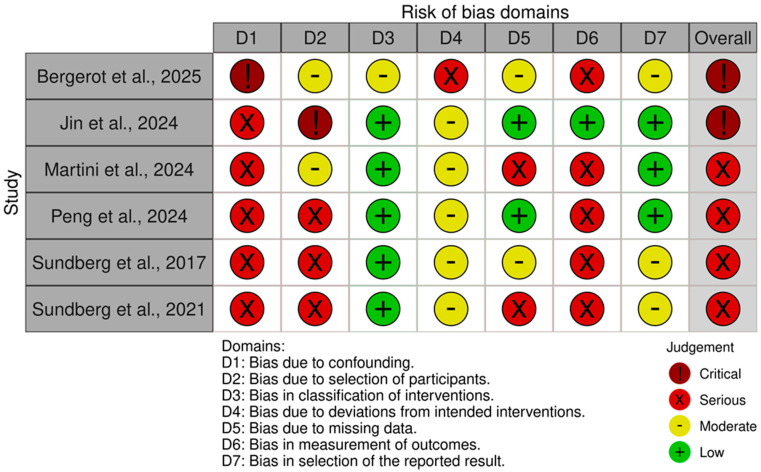
“Traffic light” plots of the domain-level judgement for each individual study evaluated with ROBINS-I [17,18,20,23,29,39].

**Figure 6:**
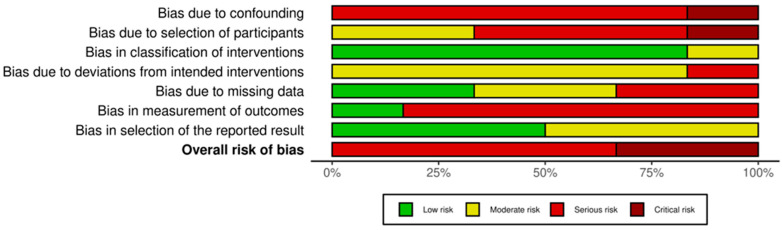
Weighted bar plots of the distribution of risk-of-bias judgements within each bias domain using ROBINS-I.

The ROBINS-I assessment for the six NRSIs revealed a higher risk of bias, as detailed in Figure 5 and Figure 6. No study was rated at ‘Low risk’. The majority (n = 4) were judged to be at ‘Serious risk’ of bias. A notable number of studies were rated at High risk’ (n = 2). The primary domains contributing to these risk levels were Domain 1 (bias due to confounding) and Domain 2 (bias in the selection of participants), indicating that many studies did not adequately control for key prognostic variables or selection factors.

## 4. Discussion

This systematic review evaluated the current evidence on the use of mobile health (mHealth) applications in uro-oncology, encompassing 29 studies that primarily focused on prostate cancer, with some on bladder and kidney cancers. The findings suggest that mHealth apps hold considerable potential to enhance patient care, but notable challenges remain. Although most evidence to date is concentrated in prostate cancer, we deliberately adopted the term ‘uro-oncology’ to reflect the full scope of interest in genitourinary malignancies, recognizing that the development of mHealth tools for bladder and kidney cancers remains underexplored and represents an important area for future research.

The included studies reflect a growing interest in the use of mobile health interventions in uro-oncology. Across the selected literature, certain trends emerge: most interventions demonstrated high patient satisfaction, improved symptom reporting and potential for integration into outpatient care. However, pronounced heterogeneity in study design, outcome measures, and target populations—alongside a predominant focus on prostate cancer—limits the generalizability and comparability of findings. These patterns suggest that while mHealth holds promise in this field, its current evidence base is fragmented and largely exploratory.

A key benefit of mHealth apps is their capacity to facilitate continuous symptom monitoring, enabling timely intervention and improved symptom management. Studies evaluating apps like Interaktor [16,18,21,27,28,34,39] demonstrated reductions in symptom burden, such as fatigue, insomnia, and urinary symptoms, in prostate cancer patients undergoing radiotherapy. Noteworthy improvements in emotional symptoms and fatigue were also reported in patients with metastatic renal cell carcinoma using a mindfulness app [23]. This real-time monitoring capability allows for early detection of adverse events, leading to improved symptom control, better treatment adherence, reduced emergency department visits, and optimized healthcare resource utilization. Furthermore, studies highlighted the value of daily symptom reporting in bladder and prostate cancer patients [21,32,34], reinforcing the role of digital tools in enhancing self-management and adherence to medical recommendations.

mHealth apps have also been shown to increase patient engagement and adherence to treatment. High compliance rates with app-based instructions and follow-up care were reported in post-operative settings, suggesting that mHealth can play a crucial role in supporting patients through complex treatment pathways [22,40]. Specific interventions improved adherence to physical activity and diet recommendations and compliance with bladder filling protocols before radiotherapy [29]. Decision-support apps, such as the CHAMPION intervention and tools like the Rotterdam Prostate Cancer Risk Calculator, have been shown to foster patient confidence in treatment decisions and aid in reducing overdiagnosis [26]. High patient satisfaction with ePRO apps further supports their role in care pathways [26].

Improving quality of life is a critical aspect of cancer care. mHealth apps have demonstrated a positive impact in this domain. Continuous feedback through apps improved emotional well-being and reduced anxiety and stress among prostate cancer patients [39]. The use of ePRO apps contributed to a better understanding of health status, which is crucial for tailoring care to individual needs [40]. Improvements in various HRQoL domains, including emotional, physical, social function, and self-care abilities, were consistently observed [20].

To our knowledge, this is the first systematic review that specifically synthesizes the role of mHealth applications across various domains of care in uro-oncology, thus offering a focused and updated overview of their current capabilities and implementation challenges.

Despite their potential, the implementation of mHealth apps faces challenges such as technical barriers and inequalities in access [10,16,36]. Initial difficulties with registration, download, or internet connection were reported [23]. Digital literacy was identified as a limiting factor, particularly among older adults [25]. Moreover, disparities in the availability and use of mHealth tools among minority and lower-income populations were highlighted, with education being a more relevant predictor than income for access to health apps [25]. Privacy and security concerns regarding sensitive data exchange also persist [11,40].

The inconsistent quality of available apps is also a concern. Amor-García et al. and La Rocca et al. noted a suboptimal average MARS score for app quality, with the “Engagement” domain often scoring lowest [10,33]. A vast portion of apps lacked interactivity, personalized, or evidence-based content. The lack of standardized evaluation criteria makes it challenging for clinicians to confidently recommend specific tools. Furthermore, adherence to clinical guidelines was often low. Skepticism from physicians regarding patients’ ability to use mHealth technology has also been reported [35].

Despite the promising results, the included studies presented several limitations that should be acknowledged. These include small sample sizes, lack of randomization in many studies (e.g., historical controls, single-arm designs), short intervention durations, absence of long-term follow-up, and limited representation of diverse populations, often focusing on highly educated or specific demographics. Furthermore, the quality and validation of the apps varied considerably, and digital literacy or access disparities were rarely addressed, potentially impacting the generalizability of findings. The reliance on self-reported data for adherence and engagement also introduces a risk of bias.

This review has revealed several critical areas where future mHealth app development in uro-oncology should be directed. Firstly, there is a clear need for apps that address a broader range of urological cancers beyond prostate cancer, including bladder, kidney, and testicular cancers. Existing apps predominantly focus on localized disease stages, leaving a notable gap in supportive care for patients with advanced or metastatic disease.

Secondly, a more holistic and integrated approach to app development is needed. Currently, most apps are disease-specific and focus on a single aspect of patient care, such as symptom tracking or medication reminders. There is a crucial opportunity to develop comprehensive apps that address multiple needs across the cancer care continuum, including symptom management, treatment adherence, patient education, and psychosocial support. The active involvement of patients and healthcare professionals, including nurses and multidisciplinary teams, in the co-design and development of apps is critical to ensure content relevance, usability, efficacy, and adherence to clinical guidelines.

Thirdly, future mHealth apps should prioritize facilitating communication and collaboration between patients and clinicians. Most existing apps are designed for either the patient or the clinician, with limited features to support shared decision-making and care coordination. Apps that promote seamless information exchange and facilitate remote monitoring could enhance the patient–clinician relationship and improve care delivery. Addressing these unmet needs will require a concerted effort from researchers, clinicians, and app developers. By focusing on broader disease coverage, comprehensive functionality, enhanced patient–clinician communication, and rigorous evaluation of user satisfaction, future mHealth apps have the potential to revolutionize uro-oncology care and improve patient outcomes.

This review provides a comprehensive overview of mHealth apps in uro-oncology, but it is essential to acknowledge its limitations. The heterogeneity of the studies makes direct comparisons challenging. Publication bias towards positive outcomes is possible. Most of the studies reviewed were short-term, and longitudinal studies are needed to assess the long-term impact of mHealth interventions. Furthermore, the small number of clinical trials and the predominance of observational studies affect the overall quality of this systematic review. Unlike previous literature reviews that predominantly focus on individual mHealth applications or a single malignancy—typically prostate cancer—our work offers a structured synthesis of mHealth interventions in the field of uro-oncology. While most available studies indeed concerned prostate cancer, we explicitly highlight this evidence gap for bladder and kidney cancers and frame it as a critical direction for future development. Additionally, our review integrates both clinical and technological outcomes and emphasizes patient-centered implementation challenges, which are often underexplored in prior literature.

While this review provides a comprehensive overview of mHealth in uro-oncology, its conclusions must be framed by the crucial methodological limitations of the primary literature. The formal risk of bias assessment, when performed, quantitatively confirms this weakness. Our analysis revealed that none of the included RCTs demonstrated a low risk of bias, with most raising ‘some concerns’ or directly at ‘high risk’. This warrants caution when interpreting the magnitude of the reported effects on symptom control and quality of life from these trials.

More critically, the evidence from non-randomized studies, which form the bulk of the literature, is substantially compromised. The finding that 100% of NRSIs were at a ‘Serious’ or ‘Critical’ risk of bias—primarily due to uncontrolled confounding and selection bias—severely limits the ability to draw causal inferences about the effectiveness of these mHealth interventions. Consequently, while the consistent reports of feasibility and patient satisfaction are valuable, the current evidence base for clinical efficacy is not robust. This underscores an urgent need for future research to prioritize rigorously designed, pre-registered RCTs with low risk of bias to definitively establish the impact of mHealth tools on patient-centered outcomes in uro-oncology.

## 5. Recommendations and Future Directions

Based on the findings and limitations of the included studies, several key recommendations can be made to advance the field of mHealth in uro-oncology. A primary imperative is to enhance the clinical validation and transparency of mHealth applications. Developers should prioritize the evaluation of these tools through high-quality clinical trials and provide comprehensive documentation of their functionalities and clinical outcomes. Currently, a notable portion of apps lacks rigorous scientific support or adherence to clinical guidelines.

Furthermore, future interventions must strategically address digital inequity by considering access disparities related to socioeconomic status, age, and digital literacy. This necessitates designing multilingual, low-bandwidth, and device-agnostic solutions that are accessible across diverse populations, including older adults and minority groups. To improve clinical utility and ensure data continuity across care settings, promoting interoperability with electronic health records (EHRs) is essential. Concurrently, the standardization of outcome measures, utilizing uniform patient-reported outcomes and validated assessment tools, would facilitate robust comparisons across studies and support future meta-analyses. The current heterogeneity in outcome measures limits generalizability.

To ensure usability, relevance, and adoption, the co-creation of these technologies—involving patients, clinicians (including nurses), and information technology specialists—should be standard practice. This collaborative approach can address issues like initial technical difficulties and lack of personalization and help maintain long-term engagement by aligning apps with evolving patient needs. Finally, the current research focus must expand beyond prostate cancer to include the development and evaluation of mHealth tools for bladder and kidney cancer populations, which are remarkably underrepresented. This expansion should also target advanced or metastatic disease stages, where a fundamental gap in supportive care currently exists.

Adopting these strategies will help bridge existing gaps in implementation, access, and evidence. This framework can guide stakeholders—including researchers, developers, and policymakers—in optimizing the transformative potential of mHealth technologies within uro-oncological care.

## 6. Conclusions

This systematic review illuminates the emerging and promising role of mobile health (mHealth) applications in uro-oncology, particularly for functions such as symptom monitoring, decision support, and the delivery of personalized care. The analyzed studies indicate predominantly positive outcomes concerning feasibility, patient engagement, and satisfaction. However, this evidence is most pronounced within prostate cancer populations, with robust data for bladder and kidney cancers remaining limited. Moreover, the methodological quality across the existing body of research is inconsistent.

These findings present critical implications for key stakeholders. For the research community, this review underscores an urgent need for rigorously designed randomized controlled trials (RCTs) with standardized outcome reporting and an expanded focus on underrepresented uro-oncological malignancies. For technology developers, the priorities should be a commitment to formal clinical validation, comprehensive usability testing, and seamless integration with existing healthcare infrastructure to ensure practical application and address issues like app quality variability and technical barriers. From a policy perspective, advancing digital inclusion initiatives and establishing clear regulatory guidance are crucial for ensuring equitable patient access and the quality assurance of mHealth applications, especially considering existing digital inequities.

Ultimately, a collaborative approach that addresses these distinct, yet interconnected, dimensions is essential for stakeholders to harness the full potential of mHealth technologies and meaningfully enhance patient-centered care in uro-oncology.

## Data Availability

The data presented in this study are available in this article.

## Abbreviations

ICT	Information and Communication Technologies
WHO	World Health Organization
eHealth	Electronic Health
mHealth	Mobile Health
Apps	Applications
PRISMA	Preferred Reporting Items for Systematic Reviews and Meta-Analyses
WoS	Web of Science
MeSH	Medical Subject Heading
RCT	Randomized Controlled Trial
RoB-2	Risk of Bias Tool 2
NRSIs	Non-Randomized Studies of Intervention
ROBINS-I	Risk of Bias in Non-Randomized Studies of Intervention
MARS	Mobile Application Rating Scale
mRCC	Metastatic Renal Cell Carcinoma
HRQoL	Health-related Quality of Life
EUC	Enhanced Usual Care
HRQL-PSY	Psychosocial Health-related Quality of Life
ePRO	Electronic Patient Reported Outcomes
QALYs	Quality-adjusted Life Years
SD	Standard Deviation
BCT	Behavior Change Technique
HADS	Hospital Anxiety and Depression Scale
MHA	Mobile Health Application
BCa	Bladder Cancer
EAU	European Association of Urology
CAFs-RGs	Cancer-associated Fibroblast-Related Genes
PCa	Prostate Cancer
BRFS	Biochemical Recurrence-free Survival
MetS	Metabolic Syndrome
SOC	Standard of Care
RPCRC	Rotterdam Prostate Cancer Risk Calculator
PRIAS	Prostate Cancer Research International Active Surveillance
RCC	Renal Cell Carcinoma
ADT	Androgen Deprivation Therapy
FBS	Fasting Blood Glucose
AC	Abdominal Circumference
RP	Radical Prostatectomy
